# Experienced versus Inexperienced Interexaminer Reliability on Location and Classification of Myofascial Trigger Point Palpation to Diagnose Lateral Epicondylalgia: An Observational Cross-Sectional Study

**DOI:** 10.1155/2016/6059719

**Published:** 2016-01-10

**Authors:** Raquel Mora-Relucio, Susana Núñez-Nagy, Tomás Gallego-Izquierdo, Alma Rus, Gustavo Plaza-Manzano, Natalia Romero-Franco, Alejandro Ferragut-Garcías, Daniel Pecos-Martín

**Affiliations:** ^1^Free Professional Practice, 28031 Madrid, Spain; ^2^Physical Therapy Department, University of Alcalá, Alcalá de Henares, 28806 Madrid, Spain; ^3^Cellular Biology Department, University of Granada, 18010 Granada, Spain; ^4^Rehabilitation and Physical Medicine Department, Medical Hydrology, Complutense University of Madrid, 28040 Madrid, Spain; ^5^Department of Nursing and Physiotherapy, University of the Balearic Islands, 07122 Palma de Mallorca, Spain

## Abstract

The purpose was to evaluate the interexaminer reliability of experienced and inexperienced examiners on location and classification of myofascial trigger points (MTrPs) in two epicondylar muscles and the association between the MTrP found and the diagnosis of lateral epicondylalgia (LE). Fifty-two pianists (some suffered LE) voluntarily participated in the study. Three physiotherapists (one inexperienced in myofascial pain) examined, located, and marked MTrPs in the extensor carpi radialis brevis (ECRB) and extensor digitorum communis (EDC) muscles. Forearms were photographed and analyzed to establish the degree of agreement on MTrPs diagnosis. Data showed 81.73% and 77.88% of agreement on MTrP classification and 85.58% and 72.12% on MTrP location between the expert evaluators for ECRB and EDC, respectively. The agreement on MTrP classification between experienced and inexperienced examiners was 54.81% and 51.92% for ECRB and 50.00% and 55.77% for EDC. Also, agreement on MTrP location was 54.81% and 60.58% for ECRB and 48.08% and 48.08% for EDC. A strong association was found between presence of relevant MTrPs, LE diagnosis, and forearm pain when the examiners were experts. The analysis of location and classification of MTrPs in the epicondylar muscles through physical examination by experienced evaluators is reliable, reproducible, and suitable for diagnosing LE.

## 1. Introduction

Lateral epicondylalgia (LE), or Tennis Elbow, is characterized by pain over or near the lateral humeral epicondyle or in the forearm extensor muscles and is associated with loss of functional activity. This condition is accompanied by tenderness upon muscle palpation, which may radiate to the forearm and increase with muscle activity [1, 2]. Several risk factors for LE development in the working population include repetitive movements of elbow and wrist for more than 2 h/day [3] due to the overuse of the forearm extensor musculature, especially the extensor carpi radialis brevis (ECRB) [4]. In this regard, professional musicians are a sensible group, given the strict training program to which they are subjected to improve their playing skills [5], with pianists being more particularly prone to suffering from musculoskeletal disorders such as LE [6, 7]. Currently, several reliable techniques are used to diagnose LE, such as elastography [8] and ultrasound [9], but they imply high costs. Therefore, the patient's symptoms are the main criteria normally used to diagnose LE. In this regard, pain evoked by palpation of the myofascial trigger points (MTrPs) in the epicondyle musculature is a key criterion for LE diagnosis.

The MTrP is a hyperirritable focal point within some tense fibers of skeletal muscle. This point is painful under compression and may cause characteristic referred pain, motor dysfunction, and autonomic phenomena [10]. A MTrP may alter muscular contraction patterns and the effectiveness of joint movements [11]. Clinically, MTrPs can be relevant (symptom-producing) or latent (not spontaneously symptomatic) [12, 13]. Relevant MTrPs evoke the referred pain described by LE patients. Although latent MTrPs are not an immediate source of pain, they may change muscle activation patterns and generate nociceptive behaviors [11]. Moreover, muscle overuse may increase the irritability of latent MTrPs [10, 11]. MTrP detection is used for the diagnosis of some musculoskeletal pain syndromes [14, 15]. In some cases, such as fibromyalgia, the presence of MTrPs is one of the main diagnostic criteria [16]. It has been demonstrated that referred pain patterns evoked by compression of MTrPs in the forearm extensor muscles reproduce the pain patterns of LE patients [10], making it useful for LE diagnosis. In this sense, the correct location of MTrPs seems to be essential for diagnosis and subsequent treatment of this pathology [1].

In clinical practice, palpation of MTrPs is affected by the manual skills of examiners. Although a standardized protocol for MTrPs palpation has been proposed by Simons et al. [10], a variety of subjective criteria may influence examiners' diagnosis. Therefore, interexaminer reliability on MTrPs palpation is required to accept it as a valid tool for LE diagnosis [17]. Since the use of innovative technologies to quantify trigger point characteristics and establish a diagnostic criterion standard test is encouraging [18] would be important to prove that the TPs palpation is a reliable and clinically relevant procedure for diagnostic and treatment of the LE.

In this regard, the purpose of this study is to evaluate the interexaminer reliability of experienced and inexperienced examiners on MTrP location and classification in the epicondyle musculature using scanned images. Moreover, associations among examiners' findings, MTrP found and LE diagnosis, and subjects' symptoms were investigated to validate MTrP manual palpation as a diagnostic tool. To the best of our knowledge, this is the first study that analyzes reliability on MTrPs palpation using the novel and complex method of image analysis. The inclusion of inexperienced and experienced examiners will be useful to determine or not the clinical experience as a prerequisite for a valid and reliable LE diagnose.

## 2. Methods

This is an observational cross-sectional study that took place at the University Hospital of the city, during October of 2014. The present study was guided by the STROBE guidelines (see Additional file in Supplementary Material available online at http://dx.doi.org/10.1155/2016/6059719).

### 2.1. Evaluators

To reduce error chance [19], three physical therapists performed the MTrP evaluation. Two were expert physiotherapists with over 10 years' experience in the diagnosis and treatment of MTrPs and the third was a physical therapist without clinical experience in myofascial pain. All examiners were blinded to LE medical diagnosis of patients.

Prior to the study, two training sessions of one hour each were performed to establish a consensus for MTrPs diagnostic criteria and to practice the procedure to be carried out. These sessions were performed to determine the time required for MTrPs assessment in each subject, as well as the most appropriate area for palpation of MTrPs. The diagnostic criteria included the following: (I) presence of a palpable taut band in a skeletal muscle; (II) presence of a hypersensitive tender spot within the taut band; (III) local twitch response elicited by a snapping palpation of the taut band; and (IV) reproduction of pain in response to MTrP compression. The MTrP was considered relevant if the pain evoked by its compression reproduced the same pain pattern in the patient's elbow and/or forearm. A MTrP was considered nonrelevant if the evoked referred pain did not reproduce a recognizable pain [1].

### 2.2. Subjects

Fifty-two subjects who were piano students or teachers were randomly recruited from the Musical Education Center “Federico Moreno Torroba” in Madrid (Spain) during September of 2014. All subjects participated voluntarily and signed an informed consent form (the form was signed by the legal representative in case of volunteers under 18 years of age). The following inclusion criteria were established: all subjects were piano students or had completed their studies but continued playing the piano. Those suffering from LE provided the medical diagnosis. The following exclusion criteria were established: shoulder pathology, cervical radiculopathy or whiplash trauma, systemic diseases, cutaneous or subcutaneous alterations in the examination area, determined by visual or palpatory evaluation, and a history of diseases in the upper extremities (previous neck pain, previous neurological disorders, or fractures in the wrist), as well as participants diagnosed with LE, prior to any surgical operation or injection of corticoids because of the anti-inflammatory effects of these injections that are believed to relieve pain and diminish disability [20].

The software “Tamaño de la muestra” version 1.1 was employed to calculate the sample size according to Cicchetti [21, 22], who reported that sample size calculation in concordance studies using the Kappa coefficient “sample size = 2*k* ^2^” (*k* = categories used in the study) is required. Since three categories were considered (0 = absent MTrPs; 1 = nonrelevant MTrPs; 2 = relevant MTrPs), the sample size required is 36 participants. After adjusting the sample size according to the percentage of losses, 45 participants were needed.

### 2.3. Materials

**Figure 1:**
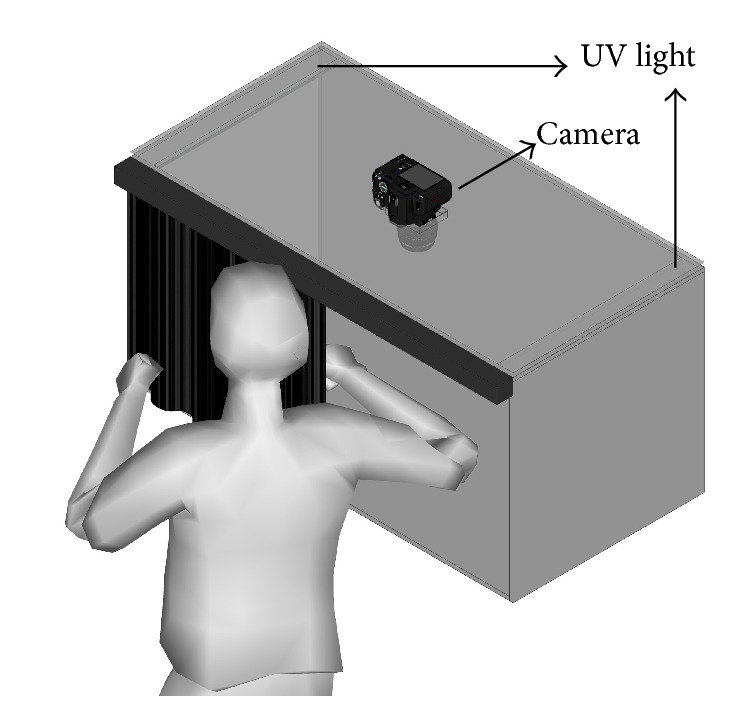
Camera obscura, UV lamps, and digital camera used to obtain images of the myofascial trigger points (MTrPs) marks made by the examiners.

Three different-colored markers for topical skin use (AINBOW, CH6004 model) were used to mark MTrPs, marking the skin with invisible and indelible ink. Each examiner used the same marker throughout the procedure. An SLR Digital Camera (Panasonic Lumix DMC-G10) was used to photograph the forearms. Two black luminescence tubes (Philips Lighting T8 18 W) were used as the UV light source (315 to 400 nm). The UV lamps were placed inside a rigid and opaque camera obscura (Figure 1). The digital camera was located over the camera obscura with the lens inward to attenuate ambient light and obtain sharp images of the MTrP marks. Three massage tables were used and the materials were not exchanged or replaced throughout the study to avoid errors during data collection.

### 2.4. MTrP Evaluation and Myofascial Pain Syndrome Diagnosis Procedure

The experimental procedure was approved by the Ethics Committee of the University Hospital (Ref. OE 29/2011).

The tested muscles were the extensor carpi radialis brevis (ECRB) and the extensor digitorum communis (EDC), given that both are easy to access and were often sore and overused in the pianists.

Each examiner and subject were given a number to preserve confidentiality. At the beginning of the session, an assessor explained the procedure to the participants and collected the medical diagnoses from those subjects with LE. This assessor did not participate in the evaluation process.

Each physical therapist examined the ECRB and EDC muscles of both forearms in all patients to determine the presence of MTrPs. Patients were in a sitting position and had the upper limbs at a 50° shoulder flexion, elbows propped on the table, the shoulder in neutral rotation, and the forearms in pronation. This procedure was always performed in the same room and under the same ambient conditions.

During the examination, the evaluators were allowed to ask the patients about presence of local or referred pain, pain intensity, and pain recurrence. No more questions were allowed. Although patients were encouraged to interact with examiners, they were not allowed to reveal the previous examiner's findings or condition of LE.

If an examiner found the MTrP in a muscle, the MTrP was classified as relevant or nonrelevant. For this, the MTrP was marked with an invisible ink marker by writing an “X” for a relevant MTrP or a dot for a nonrelevant MTrP. The use of invisible ink markers ensured that evaluators were blinded to the previous examiner's diagnosis.

Once the examination of the muscles of both forearms was completed by one of the physical therapists, the patient waited alone in the room until the next examiner came. In this way, interferences among examiners could be avoided. This waiting time of 5 min was useful in order to minimize the evidence of hypersensitivity or erythema on the examined area and to avoid a possible examiner bias. The waiting time was calculated by the assessor based on previously published data [23], as well as on the initial training sessions.

### 2.5. Picture Acquisition and Analysis

Once the procedure was completed, pictures of each patient's forearm were taken in the camera obscura. All pictures were taken by the same photographer using the same photography parameters. To take the picture, the patients adopted the same position as for the assessment. The camera was located 50 cm above the forearm, and an overhead shot was taken. Each picture included patient number and a scale bar. The UV light revealed the MTrP marks performed by the examiners.

**Figure 2:**
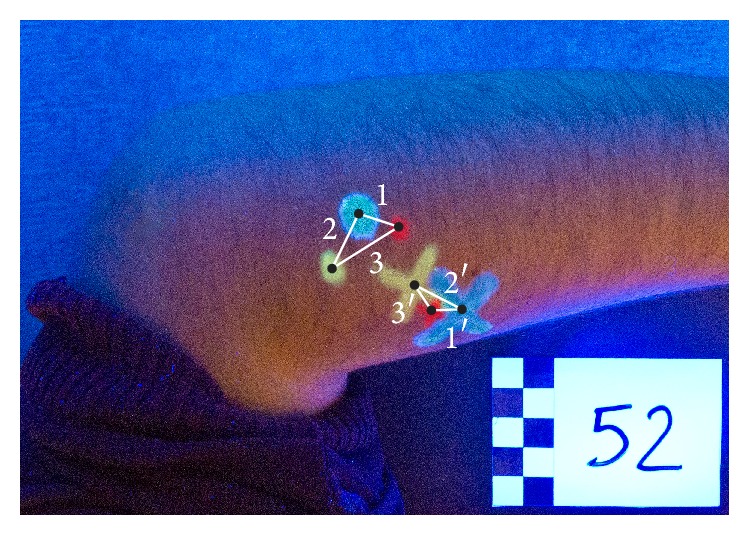
Image of the marks made by the examiners on the epicondyle muscles of each participant.

The photographs were reviewed by a different specialist, blinded to the data. The image analysis software AutoCAD 2012 was used to determine the distance between the MTrP marks made by the examiners. The midpoint between the marked MTrPs was first calculated. Using the scale bar contained in each picture as a reference, the distance between the MTrP mark made by Evaluator 1 (E.1) and by Evaluator 2 (E.2) on each muscle was determined, as well as the distance between the E.2 mark and the one made by Evaluator 3 (E.3). Lastly, the distance between the E.3 and E.1 mark was measured (Figure 2).

### 2.6. Variables Analyzed

Some categorical variables were created to analyze the agreement between evaluators:

 “MTrP classification” is a variable with three categories: Relevant MTrP, Nonrelevant MTrP, or Absent MTrP.
 “MTrP location” is a generated variable with two modalities: Agreement and Disagreement. Agreement means that the marks made by the two evaluators are placed within 1.5 cm of distance. This distance corresponds to the average width of a fingertip, according to the study of Dandekar et al. [24].
 “Complete Agreement” is a variable generated to express total agreement on MTrP location and classification. This variable has two levels: Complete agreement and Incomplete agreement. Incomplete agreement refers to a conflict between evaluators about location and/or classification of MTrPs.

### 2.7. Statistical Analysis

Management and data analysis were performed using the statistical package SPSS for Windows version 19.0 (SPSS Inc., Chicago, IL, USA). All statistical tests were calculated with a confidence interval of 95%. Each subject was considered separately.

E.1 evaluations were compared to those of E.2, and those of both experts were compared to the evaluations of E.3.

Pondered Cohen's Kappa Coefficient (quadratic weighting) test for interexaminer agreement was used to determine the agreement on MTrP classification in the ECRB and EDC muscles. This test is a descriptive statistic that measures the agreement between examiners [25]. Quadratic weighting was used because MTrP was classified into three categories (0 = MTrP does not exist; 1 = MTrP nonrelevant; 2 = MTrP relevant) and this test allows a higher ponderation about the disagreements [25], as it was important to determine the agreement between the three categories of the classification during the MTrPs location.

Cohen's Kappa Coefficient was used to establish interexaminer agreement on MTrP location and the “Complete Agreement” variable. Interexaminer agreement on MTrP location was perceived as a distance ≤1.5 cm. If the distance was >1.5 cm, diagnosis was considered to be distinct. The 95% confidence interval was included.

The number of agreements on MTrP classification, location, and “Complete Agreement” was also expressed as a percentage (number of agreements/total cases).

Cramer's V Coefficient was used to assess associations between presence of relevant MTrPs in the ECRB and/or EDC and the patient-specific variables “presence of pain in forearms” and “LE medical diagnosis.”

The degree of agreement was determined following the criteria proposed by Landis and Koch [26].

## 3. Results

### 3.1. Demographic Data

A total of 56 volunteers were interviewed for the study. Four did not meet the inclusion criteria and were excluded: two subjects had a history of wrist fracture, one subject had skin alterations on the forearm, and the other suffered from LE and had been treated with corticoids. Therefore, 52 subjects were included in the study, ranging from 8 to 61 years old (mean: 20.77, SD: 12.190).  Table 1 shows the characteristics of the study population.

### 3.2. Interexaminer Reliability on MTrP Classification

Table 2 summarizes the results of the interexaminer reliability on MTrP classification in ECRB and EDC muscles among expert (E.1, E.2) and inexperienced (E.3) examiners. These data suggested a moderate to substantial agreement on MTrP classification in ECRB muscle between experienced observers (Kappa E.1-E.2 = 0.61, 81.73% of agreement). The agreement was lower when comparing experienced and inexperienced examiners (Kappa E.1-E.3 = 0.39, 54.81% of agreement; Kappa E.2-E.3 = 0.36, 51.92% of agreement). The interexaminer reliability on MTrP classification in the EDC muscle between experienced examiners showed a moderate agreement (Kappa E.1-E.2 = 0.52, 77.88% of agreement). However, the agreement between experienced examiners and inexperienced evaluator decreased considerably (Kappa E.1-E.3 = 0.29, 50.00% of agreement; Kappa E.2-E.3 = 0.30, 55.77% of agreement).

### 3.3. Interexaminer Reliability on MTrP Location

Table 2 shows the results of interexaminer reliability on MTrP location. Data revealed a substantial agreement on MTrP location in the ECRB muscle between experienced examiners (Kappa E.1-E.2 = 0.61, 85.58% of agreement). A fair concordance between the experienced evaluators was detected in the EDC (Kappa E.1-E.2 = 0.59, 72.12% of agreement). Conversely, the reliability on MTrP location between experienced and inexperienced evaluators decreased to little accordance or absence of concordance in the ECRB (Kappa E.1-E.3 = 0.02, 54.81% of agreement; Kappa E.2-E.3 = −0.00, 60.58% of agreement) and EDC muscles (Kappa E.1-E.3 = 0.1941, 48.08% of agreement; Kappa E.2-E.3 = 0.20, 48.08% of agreement).

### 3.4. Interexaminer Reliability on “Complete Agreement”

The variable “Complete Agreement” showed different results depending on the experience of the examiners (Table 2). A moderate agreement on both MTrP classification and location in the muscles analyzed was detected between experienced evaluators (Kappa E.1-E.2 = 0.56, 70.19% of agreement in the ECRB; Kappa E.1-E.2 = 0.44, 57.69% of agreement in the EDC). Nonetheless, a fair agreement was found between expert and inexpert examiners in the ECRB (Kappa E.1-E.3 = 0.46, 46.15% of agreement; Kappa E.2-E.3 = 0.30, 45.19% of agreement) and EDC (Kappa E.1-E.3 = 0.22, 39.42% of agreement; Kappa E.2-E.3 = 0.12, 42.31% of agreement).

### 3.5. Association Analysis

Table 3 shows that the detection of relevant MTrPs in the ECRB and/or EDC by experienced examiners significantly correlated with forearm pain (E.1 = 0.54, *p* < 0.001; E.2 = 0.45, *p* < 0.001) and LE medical diagnosis (E.1 = 0.68, *p* < 0.001; E.2 = 0.60, *p* < 0.001). Contrarily, the presence of relevant MTrPs, determined by the inexperienced examiner, did not correlate with LE diagnosis (E.3 = 0.02, *p* = 0.873), nor the presence of forearm pain (E.3 = 0.123, *p* = 0.400).

## 4. Discussion

The purpose of the present study was to investigate the interexaminer reliability on location and classification of MTrP in two epicondylar muscles: the extensor carpi radialis brevis and the extensor digitorum communis muscles, which are commonly implicated [27, 28]. The study used a previously applied assessment procedure [29], as well as a novel method of image analysis to evaluate the interexaminer reliability of accurate MTrPs location and classification in the epicondyle musculature. Associations among examiners' findings, LE diagnosis, and subjects' symptoms were also investigated.

Findings suggested an acceptable agreement on MTrPs classification in both ECRB and EDC muscles between the expert examiners. Nevertheless, when comparing expert and nonexpert evaluators, the agreement level decreased dramatically. The highest agreement level found on MTrPs classification corresponded to Pondered Cohen's Kappa Coefficient values of 0.617 and 0.593 between experienced evaluators in the ECRB and EDC, respectively, which seems to be appropriate to consider when diagnosing [29]. This level of agreement was similar to that found by Myburgh et al. in patients with neck/shoulder pain [29].

To the best of our knowledge, only two studies have investigated the agreement on MTrPs location between examiners, showing different results [23, 30]. In one of the studies [23], the agreement did not exceed 21%, while the other [30] demonstrated a good clinical precision amongst experienced clinicians with a degree of concordance between 83% and 92%. These two studies were conducted in healthy subjects and evaluated the precision of manual palpation when locating nonrelevant MTrPs in the trapezius muscle by using a different methodology to the one used in the present study. These are the main problems meaning it is not possible to compare effectively the results of these works with the present study.

This study is the first to examine the interrater reliability location of relevant MTrPs by using photogrammetry measurement techniques to quantify the precision of such a location. The present study has shown an agreement on MTrP location, at a distance <1.5 cm [24, 31], among expert examiners, of 85.58% in the case of the ECRB and 72.12% for the EDC muscle. A distance of 1.5 cm was considered because of the standard size of the fingertip pulp reported by previous studies [20].

Nevertheless, when comparing MTrPs location between expert evaluators and the inexpert examiner, a strong decrease in the percentage of agreement was found, suggesting that an evaluator's manual skills may play a key role in diagnostic accuracy.

These data also showed that the percentage of accordance on “Complete Agreement” (MTrPs classification and location) decreased among the experts as well as when comparing expert and inexperienced examiners in the muscles analyzed with data concerning MTrPs classification and MTrPs location separately. However, the agreement on “Complete Agreement” among experts remained significantly higher. According to former studies [29, 32–34], our results corroborate that specific training and clinical experience are important keys for MTrPs diagnosis and, thus, to design a correct treatment protocol for patients.

Finally, the association between the presence of MTrPs and patient-specific variables was investigated. Data showed that detection of relevant MTrPs by experienced evaluators correlated with LE diagnosis and presence of forearm pain, suggesting that it is likely to reach an accurate diagnosis of LE by manual palpation of MTrPs without using complex and expensive medical techniques. Curiously, no associations were found in the case of the inexperienced examiner. These findings suggest that the use of algometry to diagnose LE may not be necessary to detect a reduction in pain thresholds, as some investigators have described [35]. In the same way, the use of other techniques, such as Doppler ultrasound [36], elastography [37], or magnetic resonance imaging (MRI), may not be indispensable as diagnostic tools for LE [38]. The present work supports the idea previously proposed [39] that palpation of MTrPs on the epicondyle musculature made by an experienced practitioner is an effective, fast, and inexpensive method to correctly diagnose LE.

This study presents some limitations: First, the findings may not be applied to other syndromes or pain conditions, except LE. Second, the number of examiners included in the study is small.

Regarding the validity of findings, these are limited to superficial forearm muscles and may not be generalized to deeper muscles like the supinator muscle. In addition, despite the acceptable evidence of reproducibility on location and classification of MTrPs, these results should be considered only for expert practitioners. Also, study participants were all pianists, which may hamper the extrapolation of results to the rest of the population. In this regard, further studies considering other populations are needed. Also, further studies may be of interest to compare physical exam results with laboratory findings and imaging tests to support the presence of MTrPs.

These findings show that the diagnosis of MTrPs in the epicondyle musculature through palpation is reliable when the evaluators are expert practitioners. This is supported also by the LE medical diagnosis and forearm pain.

## 5. Conclusions

The use of invisible ink pens and photographic analysis of data have proven to be effective tools to investigate the reliability of LE diagnosis by MTrPs palpation among examiners. The results have shown an acceptable evidence of reproducibility on classification and location of MTrPs in the epicondyle musculature among expert practitioners, while the reliability was significantly lower when comparing with an inexperienced evaluator. Findings also showed that the detection of relevant MTrPs by experienced examiners was associated with LE medical diagnosis and forearm pain, supporting the possibility of achieving a precise LE diagnosis by MTrPs palpation without needing expensive diagnosis techniques. These findings highlight the importance of the experience level of examiners in MTrP palpation for LE diagnosis. Therefore, clinical training of nonexpert physiotherapists may be suggested to improve interexaminer reliability on palpation of MTrPs and LE diagnosis accuracy.

## Supplementary Material

The supplementary file shows the checklist of items that should be included in reports of observational studies followed by the page in which these items are included in this manuscript.

## Figures and Tables

**Table 1:** Demographic and clinical characteristics of subjects (*n* = 52).

Variable	Category	Frequency	Percentage
Gender	Male	36	34.62%
	Female	68	65.38%

Piano level	Basic	30	28.85%
	Professional	50	48.08%
	Teacher	24	23.08%

Forearm pain	No	82	78.85%
	Yes	22	21.15%

Lateral epicondylalgia medical diagnosis	No	91	87.50%
	Yes	13	12.50%

Dominant hand	Right-handed	48	92.31%
	Left-handed	3	5.77%
	Ambidextrous	1	1.92%

**Table 2:** Interexaminer reliability on MTrP diagnosis.

Agreement on MTrP classification						
Evaluators	Extensor carpi radialis brevis			Extensor digitorum communis		
	Frequency	Percentage	Pondered Kappa Coefficient	Frequency	Percentage	Pondered Kappa Coefficient
E.1-E.2	85	81.73%	0.614^*∗*^	81	77.88%	0.523^*∗*^
E.1-E.3	57	54.81%	0.386^*∗*^	52	50.00%	0.289^*∗*^
E.2-E.3	54	51.92%	0.3618^*∗*^	58	55.77%	0.303^*∗*^

Agreement on MTrP location						
Evaluators	Extensor carpi radialis brevis			Extensor digitorum communis		
	Frequency	Percentage	Kappa Coefficient	Frequency	Percentage	Kappa Coefficient

E.1-E.2	89	85.58%	0.617^*∗*^	75	72.12%	0.593^*∗*^
E.1-E.3	57	54.81%	0.016^*∗*^	50	48.08%	0.194^*∗*^
E.2-E.3	63	60.58%	−0.003^*∗*^	50	48.08%	0.196^*∗*^

Complete agreement on MTrP						
Evaluators	Extensor carpi radialis brevis			Extensor digitorum communis		
	Frequency	Percentage	Kappa Coefficient	Frequency	Percentage	Kappa Coefficient

E.1-E.2	73	70.19%	0.555^*∗*^	60	57.69%	0.438^*∗*^
E.1-E.3	48	46.15%	0.455^*∗*^	41	39.42%	0.224^*∗*^
E.2-E.3	47	45.19%	0.297^*∗*^	44	42.31%	0.124^*∗*^

MTrP: myofascial trigger point; E.1 and E.2: expert evaluators on myofascial diagnosis; E.3: inexperienced evaluator on myofascial diagnosis; Frequency: frequency of agreement between evaluators; Percentage: percentage of agreement between evaluators. (^*∗*^) = *p* value < 0.001.

**Table 3:** Correlation analysis between presence of relevant MTrPs, forearm pain level, and LE medical diagnosis.

Presence of relevant MTrPs in EDC and/or ECRB	LE diagnosis Cramer's V Coefficient (*p* value)	Presence of forearm pain Cramer's V Coefficient (*p* value)
E.1	0.541 (*p* > 0.001)	0.684 (*p* > 0.001)
E.2	0.459 (*p* > 0.001)	0.606 (*p* > 0.001)
E.3	0.022 (*p* = 0.873)	0.135 (*p* = 0.400)

TrP: myofascial trigger point; E.1, E.2: expert evaluators on myofascial diagnosis; E.3: evaluator without experience on myofascial diagnosis; ECRB: extensor carpi radialis brevis; EDC: extensor digitorum communis; LE: lateral epicondylalgia; (^*∗*^) = *p* Value < 0.001.

## Abbreviations

MTrP: Myofascial trigger points
LE: Lateral epicondylalgia
EDC: Extensor digitorum communis
ECRB: Extensor carpi radialis brevis.
