# Supplementary material for: Experienced versus Inexperienced Interexaminer Reliability on Location and Classification of Myofascial Trigger Point Palpation to Diagnose Lateral Epicondylalgia: An Observational Cross-Sectional Study
Source: Evid Based Complement Alternat Med. 2016 Jan 10;2016:6059719. doi: 10.1155/2016/6059719 (PMC4736912; doi:10.1155/2016/6059719)
Supplement: Supplementary file 1 — The supplementary file shows the checklist of items that should be included in reports of observational studies followed by the page in which these items are included in this manuscript. [file 6059719.f1.doc]

Dear Editor,

Thank you for your letter. We were pleased to know that our manuscript was rated as potentially acceptable for publication in Evidence-Based Complementary and Alternative Medicine, subject to adequate revision and response to the comments raised by the reviewer.

Based on the reviewer comments, we uploaded the file of the revised manuscript on the website.

Appended to this letter is our point-by-point response to the comments raised by the reviewer. As you notice, we agreed with all the comments raised by the reviewer. Also, according to the reviewer recommendation, our manuscript has been professionally proofread to ensure its quality and avoid grammatical errors. We have added the certificate from the language editing service as supplementary material.

Another important question that we would like to ask is the possibility to modify one of the authors: with this change, Francisco Molina would be out of the manuscript, and Alma Rus would be in instead, would it be possible? The details of the new author and the definitive authors are included in the “revised manuscript” file.

We would like to take this opportunity to express our sincere thanks to the reviewer who identified areas of our manuscript that needed corrections or modification. We would like also to thank you for allowing us to resubmit a revised copy of the manuscript.

I hope that the revised manuscript is accepted for publication in Evidence-Based Complementary and Alternative Medicine.

Yours sincerely,

Daniel Pecos-Martín
